# Focal Defects of the Knee Articular Surface: Evidence of a Regenerative Potential Pattern in Osteochondritis Dissecans and Degenerative Lesions

**DOI:** 10.1155/2017/9036305

**Published:** 2017-07-09

**Authors:** Elena Gabusi, Cristina Manferdini, Francesca Paolella, Laura Gambari, Elizaveta Kon, Giuseppe Filardo, Erminia Mariani, Gina Lisignoli

**Affiliations:** ^1^SC Laboratorio di Immunoreumatologia e Rigenerazione Tissutale, Istituto Ortopedico Rizzoli, Bologna, Italy; ^2^Laboratorio RAMSES, Istituto Ortopedico Rizzoli, Bologna, Italy; ^3^Humanitas University Department of Biomedical Sciences, Via Manzoni 113, Rozzano, 20089 Milan, Italy; ^4^Humanitas Clinical and Research Center, Via Manzoni 56, Rozzano, 20089 Milan, Italy; ^5^Clinica Ortopedica e Traumatologica I, Laboratorio NABI, Istituto Ortopedico Rizzoli, Bologna, Italy; ^6^DIMEC, Alma Mater Studiorum, Università di Bologna, Bologna, Italy

## Abstract

The surgical treatment of knee articular focal lesions may offer heterogeneous clinical results. Osteochondritis dissecans (OCD) lesions showed to heal better than degenerative lesions (DL) but the underlying biological reasons are unknown. We evaluated the basal histological and immunohistochemical characteristics of these lesions analyzing a series of osteochondral fragments from young patients with similar age but presenting different etiology. Osteochondral tissue samples were stained with Safranin O and graded using a histological score. Markers of mesenchymal progenitor cells (CD146), osteoclasts (tartrate-resistant acid phosphatase, TRAP), and vessels (CD34) were evaluated. Histological score showed a higher degeneration of both cartilage and bone compartments in OCD compared to DL fragments. Only CD146-positive cells were found at the same percentage in cartilage compartment of both DL and OCD patients. By contrast, in the bone compartment a significantly higher percentage of CD146, TRAP, and CD34 markers was found in OCD compared to DL patients. These data showed distinct histological characteristics of osteochondral focal lesions located in the same anatomical region but having a different etiology. The higher percentages of these markers in OCD than in DL, mainly associated with a high bone turnover, could help to explain the higher clinical healing potential of OCD patients.

## 1. Introduction

Focal defects of the knee articular defect surface are diagnosed in 5–10% of patients undergoing knee arthroscopy [1]. Irrespective of their etiology, these lesions contribute to disability, impairing the quality of life mainly by limiting patients' social activities, and premature development of osteoarthritis (OA) [2, 3].

Various techniques, both palliative and reparative, have been used to treat this pathology with variable success rates [4]. Autograft transfer has been partially successful in reducing pain and increasing mobility, but satisfactory results have been shown only in small lesions [5]. Treatment options directed to the recruitment of bone marrow cells to obtain potential cartilage precursors have been developed to allow stem cells migration from the marrow cavity to the fibrin clot of the defect [4]. However, these treatment options such as abrasion, drilling, and microfracture produce predominantly a fibrous repair tissue which lacks the biomechanical and viscoelastic characteristics of normal hyaline cartilage [2, 6]. Moreover, these techniques target only the chondral surface, while there is an increasing awareness on the importance of considering the entire osteochondral unit [7]. In fact, the subchondral bone is involved in the etiopathological process not only primarily, like in osteochondritis dissecans (OCD), but also secondarily in degenerative lesions (DL) which have been previously considered only in terms of the depth of chondral surface involvement.

In this light, osteochondral biomaterials have been developed to restore both cartilage and the underlying subchondral bone, with encouraging clinical results [8, 9]. However, the outcome is heterogeneous and rather unpredictable [9–11]. Understanding the etiopathological patterns leading to the alteration of the articular tissues might shed some light into the healing potential of such lesions, explaining the heterogeneous results observed in osteochondral regeneration and giving possible indications to better target and further develop treatments in the future [11–13]. An accurate knowledge of cellular osteochondral tissue patterns is mandatory to improve current therapies as well as to contribute to increasing the knowledge concerning different etiologies.

CD146, a transmembrane glycoprotein, is a marker which identifies MSCs progenitor cells subpopulation present in the bone tissue [14]. CD146-positive cells have been shown to have the ability to function as self-renewing, clonogenic skeletal progenitors and to define the anatomical identity of MSC in human bone marrow [15]. Moreover, CD146-positive cells were also identified in human bone marrow in situ, on adventitial reticular cells located abluminally covering and stabilizing blood vessels, but not on erythroid or myeloid cells, endothelial cells, adipocytes, osteoblasts, osteocytes, or endosteal cells [15, 16].

CD146 is markedly downregulated during ex vivo chondrogenesis of MSC [17] and absent in normal cartilage [15]. However, the presence of MSCs in late stage OA cartilage has been reported [18].

Tartrate-resistant acid phosphatase (TRAP) is a marker used to evaluate how cells participate in the resorption of the cartilage matrix or mineralized bone matrix [19], highly expressed in polynucleated osteoclasts and chondroclasts [20].

Vascularization is another parameter that has been evaluated in several studies to establish the level of remodeling of bone/cartilage tissue using CD34 or factor VIII or VEGF as markers of endothelial cells [21, 22].

Therefore, aim of the study is to investigate the osteochondral tissue of patients, either with primary or with secondary subchondral bone involvement, in order to understand possible biological reasons for the different regenerative potential of these patients as reported in the literature.

## 2. Materials and Methods

### 2.1. Patient Characteristics

The patients included in the study presented focal lesions of the articular surface in otherwise healthy joints (no evidence of other chondral-osteochondral, ligament, meniscus, or synovial lesions), with stable and physiologically aligned knees. X-ray and MRI surgical indication were confirmed intra-articularly, when the disease was defined and staged as grade 3 focal cartilage DL in 7 patients and grade 3 OCD lesions in 7 patients, according to the ICRS evaluation package [https://www.secot.es/uploads/descargas/formacion/escalas_valoracion/ICRS._TRAUMA_CARTaILAGO.pdf] (Table 1). OCD lesions were intraoperatively found unsuitable for fragment refixation; therefore the lesion area was removed and evaluated for the current study, while the defect was reconstructed with the implantation of an osteochondral scaffold. In light of the recent evidence on the involvement of the subchondral bone also in the cartilage DL, all the 14 patients were treated with osteochondral biomaterial allowing us to retrieve osteochondral fragments during the preparation of the area for the scaffold implantation to restore the osteochondral unit.

### 2.2. Histochemical Analysis and Scoring

Osteochondral fragments were fixed in a freshly prepared 9 : 1 mixture of B5 solution (mercuric-chloride containing fixative)/40% formaldehyde and embedded in paraffin as described [23]. At least three different osteochondral fragments were collected from each patient and embedded in three different blocks. Serial sections (each 5 *µ*m thick) were cut from each block, in order to evaluate approximately 100 *µ*m of the fragment. At least 5 sections (*N* = 15 total sections analyzed for each patient) were stained with Safranin O fast green. The sections were scored by two readers (EG and GL, with experience in histological scoring, giving a single result for each scoring on a consensus basis) blinded for the patient clinical diagnosis. Intrareader variability was obtained on examination of 15 randomly selected sections (after one month) showing excellent results [total histological score in each section intraclass correlation coefficient (ICC) = 0.987, 95% CI 0.974–0.994].

We developed a histological score by integrating the usual cartilage scoring (Mankin score [24]) with a new subchondral bone scoring (Table 2) that included an evaluation of both bone trabecular structure and bone marrow. The total histological score was 26 points (maximum 16 points for cartilage and maximum 10 points for the bone). Scores from 0 to 26 points indicated not degenerated (normal, score 0) to highly degenerated osteochondral tissue (score 26). Moreover, we analyzed cartilage and a bone score separately to discriminate the degree of lesions in the two compartments.

The maximum cartilage score was 16 and we considered arbitrarily a score of <4 points as low (L); 5–9 points as medium (M); and >9 points as high (H) degenerated cartilage area. The maximum bone score was 10 and we considered arbitrarily a score of <3 points as L; 4–7 points as M; >7 points as H degenerated bone area.

### 2.3. Immunohistochemical Analysis

Histological sections were deparaffinized and incubated with monoclonal anti-human CD146 diluted 1 : 100 (clone N1238; Novocastra, Leica Biosystems, Newcastle, United Kingdom) or with mouse anti-human TRAP diluted 1 : 50 (clone 26E5; Novocastra) or with mouse anti-human CD34 (clone QBEnd 10, DakoCytomation, Glostrup, Denmark) diluted 1 : 50 in TBS containing 0.25% BSA, 0.1% NaN_3_, at room temperature for 1 hour. The slides were developed as described [25]. Negative controls were performed by omitting the primary antibody, and isotype-matched controls were performed by using an isotype-matched primary antibody.

Semiquantitative analysis of immunohistochemistry stained slides was performed on fifteen microscopic fields (20x objective lens) for each section. The analysis was performed using Red/Green/Blue (RGB) with Software NIS-Elements and Eclipse 90i microscope (Nikon Instruments Europe BV) equipped with a CCD camera (dimension of the sensor 2/3 inches) mounted on 0.7x C-mount. Briefly, we acquired the total number of blue-stained nuclei and the total number of positive-stained red cells in each field (358 × 269.15 *µ*m). The data were expressed as mean percentage of positive cells for CD146 and TRAP, respectively. The number of positive CD34 vessels was manually counted and expressed as the mean number of vessels/area. All data obtained from each fragment/section were then expressed as the median and 10th–90th percentiles.

### 2.4. Statistical Analysis

Statistical analysis was performed using nonparametric tests since the data in the OCD and DL groups did not display a normal and symmetric distribution (univariate Mann–Whitney *U* test for unpaired two-group data, univariate Jonckheere-Terpstra test for ordered data trend, and multivariate generalized linear model with gamma distribution and log-link function). CSS Statistical Software (StatSoft Inc., Tulsa, OK, USA) was used for analysis. For all tests, values of *p* < 0.05 were considered significant.

Intraclass correlation coefficient (ICC) was measured to assess the intrareader reliability for the histological scores. Values > 0.8 were considered excellent.

## 3. Results

### 3.1. Histochemical Characterization of Focal Osteochondral Fragments

**Figure 1:**
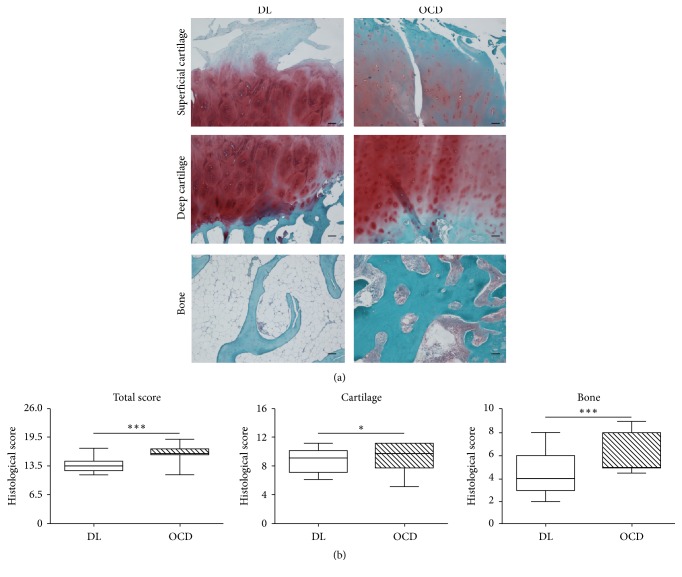
Safranin O staining on focal osteochondral samples from DL and OCD. (a) Representative Safranin O stained section of focal osteochondral lesion from DL and OCD. Bars 100 *μ*m. (b) Total histological score and cartilage and bone score of DL (number of fragments/sections = 21/63) and OCD (number of fragments/sections = 22/66). Data are expressed as median with 10th–90th percentile (whiskers). ^*∗*^*p* < 0.01 and ^*∗∗∗*^*p* < 0.0001.

In order to first characterize the degenerated areas in OCD compared to DL patients, we performed a histological analysis. In both OCD (71%) and DL (57%) young patients with similar age and BMI, the lesions occurred preferentially in medial femoral condyle (MFC) rather than lateral femoral condyle (LFC) (Table 1). When osteochondral lesions were analyzed on Safranin O stained sections (Figure 1(a)), the histological score revealed higher degenerated areas in OCD compared to DL (*p* < 0.0001; Figure 1(b), total score). Subsequently, cartilage and bone scores were considered separately and we evidenced higher lesions in OCD both in cartilage (Figure 1(b); *p* < 0.01) and in bone (Figure 1(b); *p* < 0.0001) compartments.

### 3.2. Immunohistochemical Analysis for MSCs

**Figure 2:**
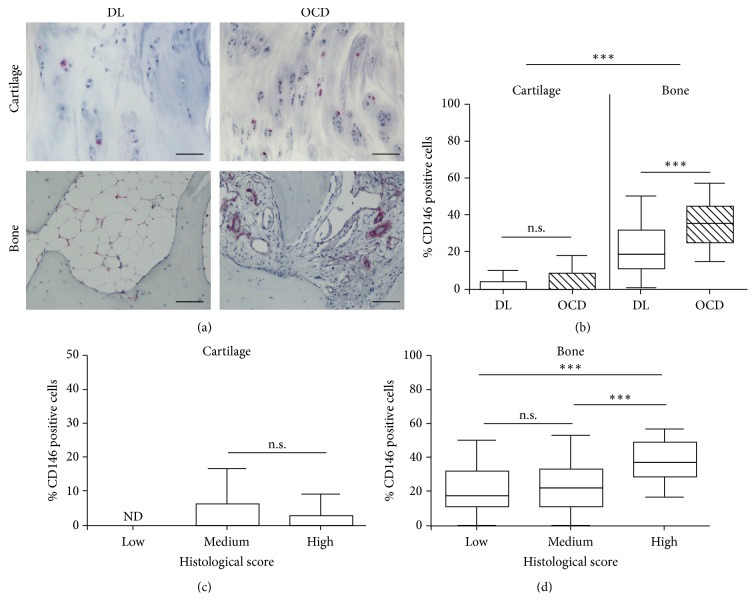
CD146 on focal osteochondral samples from DL and OCD. (a) Representative CD146 immunostained section of focal osteochondral lesion from DL and OCD. Bars 100 *μ*m. (b) Percentage of CD146-positive cells in cartilage and bone compartment of DL (number of fragments/sections = 21/21) and OCD (number of fragments/sections = 22/22). Data are expressed as median with 10th–90th percentile (whiskers). ^*∗∗∗*^*p* < 0.0005. (c) Percentage of CD146-positive cells in cartilage associated with histological score level (low, medium, and high). Data are expressed as median with 10th–90th percentile (whiskers). n.s. = not significant; ND = not detected. (d) Percentage of CD146-positive cells in bone associated with histological score level (low, medium, and high). Data are expressed as median with 10th–90th percentile (whiskers). ^*∗∗∗*^*p* < 0.00001; n.s. = not significant.

To define osteochondral tissue remodeling in focal lesions, MSCs were evaluated both in cartilage and in bone tissues by anti-CD146. In the cartilage of both DL and OCD patients we found CD146-positive cells mainly localized in the intermediate/superficial zone (Figure 2(a)). In the bone tissue of both DL and OCD patients, we observed CD146-positive cells in the bone marrow area around endothelial cells and adipocytes, while we did not find any positive staining in the trabeculae (Figure 2(a) and supplemental Figure 1, in Supplementary Material available online at https://doi.org/10.1155/2017/9036305).

We analyzed the percentage of positive CD146 cells by the multivariate generalized linear model to evaluate the combined effects of tissue area (bone and cartilage) and etiology (DL and OCD). As shown in Figure 2(b), we found that the percentage of CD146-positive cells was significantly higher in bone than in cartilage (*p* < 0.0005), independently of the etiology. Moreover, the multivariate analysis evidenced that OCD showed a significantly higher percentage of CD146-positive cells (*p* < 0.0005) compared to DL. Subsequently, in the multivariate analysis we also considered the effect of the histological score (graded as low, medium, or high) and we found that the percentage of CD146-positive cells was significantly strictly dependent on the histological score (*p* < 0.02) but not on the etiology. In particular, in the cartilage, the histological score was not significantly dependent on the percentage of positive CD146 cells (Figure 2(c)). Conversely, in bone, the percentage of CD146-positive cells was dependent on the histological score and significantly increased in high score bone tissue (*p* < 0.0001), whereas no differences were observed between low and medium scores (Figure 2(d)).

### 3.3. Immunohistochemical Analysis of Osteoclast/Chondroclast Cells

**Figure 3:**
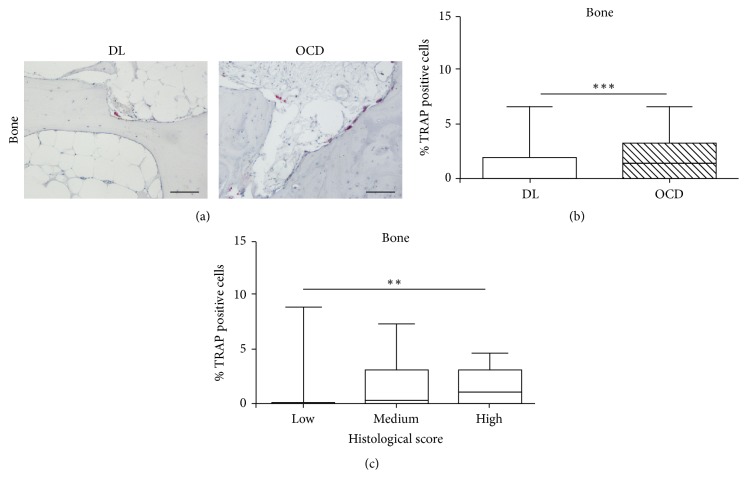
TRAP on focal osteochondral samples from DL and OCD. (a) Representative TRAP immunostained section of focal osteochondral lesion from DL and OCD. Bars 100 *μ*m. (b) Percentage of TRAP-positive cells in bone compartment of DL (number of fragments/sections = 21/21) and OCD (number of fragments/sections = 22/22). Data are expressed as median with 10th–90th percentile (whiskers). ^*∗∗∗*^*p* < 0.0001. (c) Percentage of TRAP-positive cells in bone associated with histological score level (low, medium, and high). Data are expressed as median with 10th–90th percentile (whiskers). ^*∗∗*^*p* < 0.005.

To detect the presence of bone or cartilage resorption, chondroclasts/osteoclasts were evaluated both in cartilage and in bone tissue by anti-TRAP staining. In cartilage, no TRAP-positive cells were found, either in DL or in OCD (data not shown). In bone tissue of both patient groups, TRAP-positive cells were mainly located in subchondral bone surface, near the resorption area, and frequently located underneath the articular cartilage (Figure 3(a)). Mann–Whitney analysis showed that the percentage of TRAP-positive cells was significantly higher (*p* < 0.0001) in the bone marrow of OCD than in DL (Figure 3(b)). We then considered the effect of the histological score using the test of Jonckheere-Terpstra and we found a significant positive increased trend (*p* < 0.005) of the percentage of TRAP-positive cells (Figure 3(c)) from low to high histological score. Finally, we performed the generalized linear analysis on the percentage of TRAP-positive cells with both etiology and histological score as fixed effect and we confirmed that only etiology (DL, OCD) influenced the percentage of TRAP-positive cells.

### 3.4. Immunohistochemical Analysis of Vascularization

**Figure 4:**
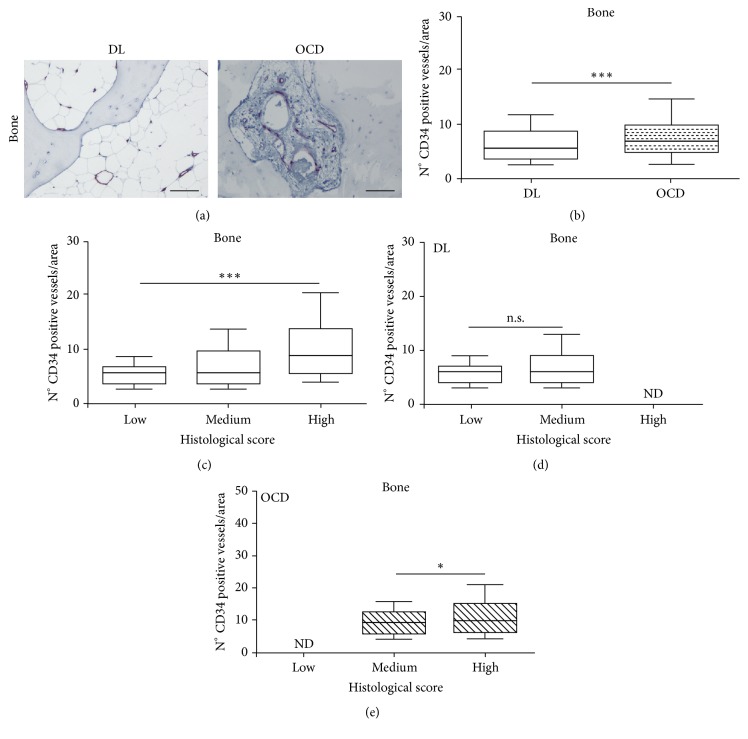
CD34 on focal osteochondral samples from DL and OCD. (a) Representative CD34 immunostained section of focal osteochondral lesion from DL and OCD. Bars 100 *μ*m. (b) Number of CD34 positive cells/area in bone compartment of DL (number of fragments/sections = 21/21) and OCD (number of fragments/sections = 22/22). Data are expressed as median with 10th–90th percentile (whiskers). ^*∗∗∗*^*p* < 0.0001. (c) Number of CD34 positive cells/area in bone associated with histological score level (low, medium, and high). Data are expressed as median with interquartile range. ^*∗∗∗*^*p* < 0.0005. (d) Number of CD34 positive cells/area in bone of DL patients associated with histological score level (low, medium, and high). Data are expressed as median with interquartile range. n.s. = not significant. (e) Number of CD34 positive cells/area in bone of OCD associated with histological score level (low, medium, and high). Data are expressed as median with interquartile range. ^*∗*^*p* < 0.05.

The number of vessels was evaluated with CD34, a typical marker of endothelial cells. We found positive vessels located underneath the tidemark in the subchondral bone (Figure 4(a) and supplemental Figure 1). The Mann–Whitney analysis showed that the percentage of CD34-positive vessels was significantly higher (*p* < 0.0001) in the bone marrow of OCD compared to DL (Figure 4(b)). Then, we considered the effect of the histological score by using the Jonckheere-Terpstra test and found a significant positively increased trend (*p* < 0.0005) of the percentage of CD34-positive vessels (Figure 4(c)) from a low to a high histological score. Finally, we performed the generalized linear analysis on the percentage of CD34-positive vessels by considering both etiologies and histological scores as fixed effects and confirmed that both etiology (*p* < 0.0005) and histological score (*p* < 0.008) influenced the percentage of CD34-positive vessels (Figures 4(b) and 4(c)). Interestingly, in DL the number of positive CD34 vessels was not significantly different between low and medium histological scores (Figure 4(d)). Conversely, in OCD the number of positive CD34 vessels was significantly increased in high scores rather than in medium scores (*p* < 0.05) (Figure 4(e)).

## 4. Discussion

The main finding of this study is that the osteochondral tissue from focal lesions, located in the same anatomical region and in patients with similar age and BMI, presented distinct histological and biologic characteristics according to the etiology, which could explain the different healing potential and the different clinical results reported in the literature treating OCD and DL with osteochondral scaffolds.

In this study, we demonstrated that focal osteochondral fragments from OCD patients present distinctive features compared to DL patients and this may explain the high healing clinical potential of OCD. To have a clear picture of the basal biological characteristics of focal osteochondral fragments removed from patients with DL and OCD, we firstly developed a histological scoring method, which allowed taking into consideration the full thickness of both cartilage and bone compartments. We then evaluated the percentage of MSCs and osteoclasts and the number of vessels, to understand the biologic changes and healing potential of these lesions.

This study showed a significantly worse histological score in OCD compared to DL both in the bone and in the cartilage compartments of young patients (with similar age and BMI), thus confirming that these focal lesions have peculiar histological characteristics with more abnormalities. In fact, we found that CD146, a specific marker of skeletal mesenchymal stem cells [14], showed a significantly higher percentage in bone compared to cartilage compartment in both DL and OCD groups and a higher percentage in the bone compartment of OCD compared to DL, which suggests a different remodeling rate in these two tissues and types of focal lesions. The percentage of MSCs increased from a lower to a worse bone histological score, independently of the etiology, thus showing that the progression of bone modifications was associated with the increase in progenitor cells. Interestingly, we found MSCs in the focal lesions of both OCD and DL cartilage. It is known that healthy cartilage is negative to the CD146 marker [15] which, on the other hand, is expressed in a chondrocytes subpopulation in the later stage of OA [18]. However, the percentage of CD146 cells in both focal lesions of DL and OCD did not increase compared to the histological score, in contrast to data found in late stage OA chondrocytes [18].

TRAP-positive osteoclasts were found only in the bone compartment of both DL and OCD patients revealing only area of bone remodeling in these focal osteochondral lesions. However, the OCD group showed a significantly higher percentage of TRAP-positive cells. These data are in line with studies by Krause et al. [3] and Yonetani et al. [26] who also showed viable subchondral trabeculae in juvenile OCD lesions arthroscopically classified according to the International Cartilage Repair Society (ICRS) OCD as ICRS OCD I/II [27]. A recent paper described a greater presence of TRAP-positive cells in sclerotic bone than in nonsclerotic bone areas, which is a typical feature in advanced OA [28], suggesting that the presence of TRAP-positive cells in bone focal lesion may contribute to a sclerotic bone evolution.

We found an increased number of vessels in OCD compared to DL, related, in both groups, to worse histological bone score as already found for CD146, which was positive on MSCs located around the endothelial vessel cells [14]. The number of vessels positive for CD34 was associated with the etiologies (DL and OCD) with a specific pattern. Interestingly, vessels were limited to the bone compartment and did not pass through the tidemark in any of the cases analyzed, which is instead a typical feature found in OA patients [29].

## 5. Conclusions

In conclusion, in this study immunohistological characterization of focal osteochondral lesions from subjects with DL or OCD allowed identifying specific remodeling patterns. Even though OCD showed overall worse scores, confirmed by the separate analysis of bone and cartilage compartments, the higher level of abnormalities was counterbalanced by higher remodeling features, which may help explaining the more successful outcome documented in the literature on OCD treatments compared to DL [11, 13]. In fact, osteochondral treatments for focal lesions of the articular surface of similar anatomic locations showed a better outcome in young adults with OCD, likely due to the higher remodeling potential documented in this study in terms of both cell composition and vascularization.

The success of a therapy might be directly dependent on the histopathological basal characteristics of the focal osteochondral lesion, and future studies should focus on the identification of further tissue characteristics and etiopathology patterns, to develop better treatment for restoring lesions of the articular surface.

## Supplementary Material

Supplemental Fig.1: CD146 and CD34 serial immunostained sections of a representative focal osteochondral lesion. Magnification 600X.

## Figures and Tables

**Table 1:** Patient characteristics.

Patients	Age	Sex	BMI	Lesion site	Diagnosis
1	30	M	23	MFC	DL
2	20	M	22	LFC	DL
3	17	M	19	LFC	DL
4	31	M	27	LFC	DL
5	23	M	24	MFC	DL
6	22	M	24	MFC	DL
7	33	M	25	MFC	DL
8	18	F	23	LFC	OCD
9	30	M	26	MFC	OCD
10	32	M	27	MFC	OCD
11	20	F	20	MFC	OCD
12	23	M	22	LFC	OCD
13	22	M	30	MFC	OCD
14	28	M	25	MFC	OCD

MFC = medial femoral condyle; LFC = lateral femoral condyle; DL = degenerative lesion; OCD = osteochondritis dissecans.

**Table 2:** Histological score.

Cartilage compartment (Mankin score [24])	
*Cartilage structure*	
Normal	0
Surface irregularities	1
Pannus and Surface irregularities	2
Clefts to transitional zone	3
Clefts to radial zone	4
Clefts to calcified zone	5
Complete disorganization	6
*Cells*	
Normal	0
Diffuse hypercellularity	1
Cloning	2
Hypocellularity	3
*Safranin O staining*	
Normal	0
Slight reduction	1
Moderate reduction	2
Severe Reduction	3
No dye noted	4
*Tidemark integrity*	
Intact	0
Crossed by blood vessels	1
Crossed by fibrous tissues	2
Duplication	3

*Bone compartment*	

*Bone plate integrity*	
Normal	0
Decreased plate thickness	1
Increased plate thickness	2
*Trabecular bone integrity*	
Normal	0
Decreased trabecular thickness	1
Increased trabecular thickness	2
*Bone cells*	
Normal cellularity	0
Hypercellularity	1
Hypocellularity	2
Empty lacunae ≥30%	3
*Bone marrow (BM) integrity*	
Normal	0
BM + with vascular component	1
BM + vascular + inflammatory cells	2
BM + vascular component + inflammatory cells + fibrous tissues ≥30%	3

Cartilage maximal score 16 (low <4; medium 5–9; high >9 score); bone maximal score 10 (low <3; medium 4–7; high >7 score); total score 26 (normal = 0).
