# Supplementary figures and images for: Focal Defects of the Knee Articular Surface: Evidence of a Regenerative Potential Pattern in Osteochondritis Dissecans and Degenerative Lesions
Source: Biomed Res Int. 2017 Jul 9;2017:9036305. doi: 10.1155/2017/9036305 (PMC5523180; doi:10.1155/2017/9036305)

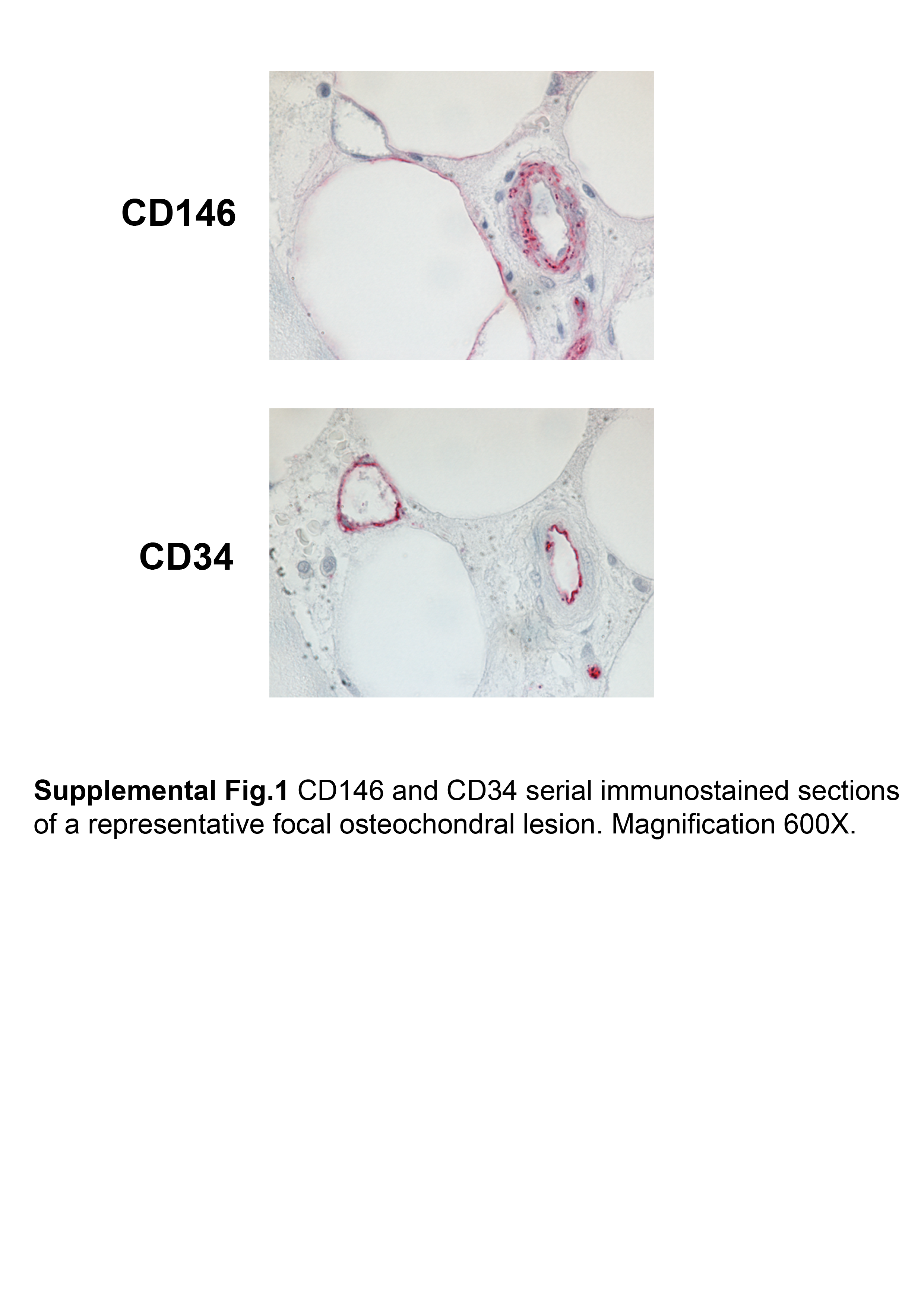

Supplement: Supplementary file 1 — Supplemental Fig.1: CD146 and CD34 serial immunostained sections of a representative focal osteochondral lesion. Magnification 600X. [file 9036305.f1.tif]
